# A time to reflect: deviations from the balanced time perspective are associated with hypomentalization

**DOI:** 10.3389/fpsyg.2024.1379585

**Published:** 2024-05-27

**Authors:** Anne Winquist, Michael Rönnlund

**Affiliations:** Department of Psychology, Umeå University, Umeå, Sweden

**Keywords:** time perspective, past, present, future, deviations from a balanced time perspective, mentalization, hypomentalization

## Abstract

**Introduction:**

Poor mentalization, or lack of capacity to reflect on self and others in terms mental states, thoughts, and feelings, and time perspective biases were both related to mental disorders and lower wellbeing in separate studies. Expanding one prior study, we examined the relationship of mentalization and time perspective, including a measure known as deviations from the balanced time perspective (DBTP) that summarizes time perspective biases across the past, present, and future time frames.

**Method:**

A convenience sample of 258 participants responded to a version of the Reflective Functioning Questionnaire (RFQ-8) and a six-dimensional version of the Zimbardo Time Perspective Inventory (S-ZTPI). Given recent evidence that the original two-factor structure of the RFQ may need to be reconsidered, we used confirmatory factor analyses (CFA) to compare alternative models for RFQ as a first step.

**Results:**

In line with several recent studies, the CFA favored a unitary model of RFQ-8 reflecting hypomentalization (or uncertainty). The total score showed significant associations with Past Negative, Present Fatalistic, and Future Negative dimensions of S-ZTPI, while hypomentalization was negatively associated with Future Positive. Of major interest, DBTP and hypomentalization showed a strong positive correlation (*r* = 0.64 for latent constructs; *r* = 0.62 in an adjusted model).

**Conclusion:**

Deviations from the balanced time perspective were substantially related to hypomentalization. Further research is required to examine the generalizability of the finding (e.g., to measures of mentalization focused on others) and to provide a better understanding of the theoretical basis of the link. Potentially shared associations in development (e.g., attachment style) and mindfulness, that may influence both time perspective and mentalization is of interest in this regard.

## Introduction

The last decade has seen a surge in studies on time perspective, the way individuals perceive and relate to the past, present, and future (Lewin, 1951; Zimbardo and Boyd, 1999), including its role in accounting for variations in behaviors and well-being, and its relations to other psychological constructs. The goal of the present study was to examine relations between time perspective and a key concept in social cognition, namely mentalization (Allen and Fonagy, 2007), a potential link that has received little attention so far.

### Time perspective

The most frequently used measure of time perspective is the Zimbardo Time Perspective Inventory (ZTPI, Zimbardo and Boyd, 1999). ZTPI include five subscales, two that concern the past, two that involve the present, and one scale that targets the future. Past Positive reflects a warm and nostalgic view of the past (e.g., *Familiar childhood sights, sounds, and smells often bring back a flood of wonderful memories*), whereas Past Negative reflects an aversive view towards the past (e.g., *I think about the bad things that have happened to me in the past*). Present Hedonistic captures a live-for-the-moment attitude toward the present with little concern for the future (e.g., *I feel that it’s more important to enjoy what you are doing than to get work done on time*), whereas Present Fatalistic reflects an attitude toward the present characterized by an external locus of control (e.g., *Since whatever will be will be, it does not really matter what I do*). Finally, Future involves an attitude and direction toward the future that involves planning and striving for rewards (e.g., *When I want to achieve something, I set goals and consider specific means for reaching those goals*). In analogy with the division of the past into positive and negative dimensions, Carelli et al. (2011) expanded the original scale to include separate Future Positive and Future Negative subscales. Future Positive is basically the same as the original Future scale (except for one item), whereas the new Future Negative scale captures an aversive attitude to the future (e.g.*, To think of the future makes me sad*). The distinction between positive and negative future dimensions was supported by several findings. For example, scores on Future Negative, but not Future Positive, were associated with maladaptive coping strategies like substance use and denial in teenagers (Blomgren et al., 2016), and levels of stress were strongly associated with Future Negative but unrelated to Future Positive (Rönnlund et al., 2018).

A basic tenet of the time perspective theory by Zimbardo and Boyd (1999, 2008) is that individuals often develop temporal biases in the form of an overfocus on a particular temporal frame or attitude (e.g., on negative aspects of the past). These biases act as a disposition that exerts an enduring influence on thoughts, feelings, and behaviors, and such time perspective biases are predictive of various kinds of behavioral problems and forms of ill-health. By contrast, the balanced time perspective (BTP) was proposed as an ideal time perspective that involves the ability to adaptively switch between temporal frames depending on situational factors and should be associated with high levels of wellbeing. BTP was thought to concur with a ZTPI score profile characterized by high scores on Past Positive, low scores on Past Negative and Present Fatalistic, a moderate score on Present Hedonistic, and a moderately high score on Future (Zimbardo and Boyd, 2008). Based on this idea, a measure known as Deviations from the Balanced Time Perspective (DBTP) was developed to measure an individuals’ distances to the BTP-profile, taking all five (Stolarski et al., 2011) or six (Rönnlund et al., 2017) ZTPI dimensions into account. This measure has now been considered in many recent studies indicating that the notion of BTP is a vital mechanism of adaption in different areas of functioning (see Stolarski et al., 2020 for a review).

### Mentalization and time perspective

Another important function for the understanding of our sense of self is mentalization. Mentalization is defined as the capacity to interpret self and others in terms of internal mental states such as wishes, feelings, intentions, and desires (Fonagy et al., 2016), often described as “the ability to see others from the inside and ourselves from the outside” (Bateman and Fonagy, 2016).

Mentalization is considered to be organized around four dimensions or polarities (Luyten et al., 2020): automatic vs. controlled mentalization (e.g., mentalizing either occurs automatically without conscious effort or is controlled by making conscious decisions to reflect on a given situation), inner vs. outer mentalization (e.g., reflection on either inner states such as emotions or thoughts, or outer states such as visible actions and behaviors), self vs. others (e.g., reflection on either one’s own feelings or thoughts, or other people’s feelings and thoughts), and cognitive vs. affective mentalization (e.g., reflection on either cognitive or emotional content).

Whereas good mentalization capabilities are linked to favorable outcomes, poor mentalization has been linked to poorer life satisfaction and a range of psychological disorders such as borderline personality disorder (Perroud et al., 2017), depression (Fischer-Kern and Tmej, 2019), anxiety disorders (Sloover et al., 2022), schizophrenia (Chung et al., 2014), and attention deficit hyperactivity disorder (ADHD; Perroud et al., 2017).

Interestingly, similar associations were observed for time perspective biases, such that greater deviations from optimal values on specific ZTPI facets and/or greater DBTP scores compared with healthy participants, were observed in individuals with anxiety disorders (Åström et al., 2019), major depressive disorder (Pyszkowska et al., 2024), schizophrenia (Styła et al., 2019), and ADHD (Carelli and Wiberg, 2012). A significant negative association of DBTP and life satisfaction was furthermore reported in several studies (e.g., Stolarski et al., 2016, 2020; Rönnlund et al., 2017).

One basis for expecting mentalization capabilities to covary with time perspective are common roots in attachment processes and early social development. In developmental models of mentalization (Luyten et al., 2020), interactions with primary caregivers are considered one essential factor. More specifically, parents with better capabilities for mentalization are more likely sensitive to their child’s needs, promoting secure attachment and epistemic trust. This increases chances of developing mentalizing capabilities further via social learning, a virtuous circle that may increase resilience to adversity and stress. One could speculate that forming a more balanced time perspective, which is related to lower stress (e.g., Papastamatelou et al., 2015; Rönnlund et al., 2018) is part of this process, as temporal perspective is an inherent part actions and events involving self and others. Conversely, once established, time biases could potentially hinder mentalization processes. For example, individuals with a marked bias toward negative aspects of the past might project past experiences onto present situations, and to show less flexibility in adapting to new information in changing social dynamics, leading to misunderstandings or misinterpretations of others’ intentions or emotions.

In any case, there is evidence based on children and adolescents that secure attachment is associated with better capabilities for mentalization, whereas forms of insecure attachment are associated with difficulties in this regard (for a review, see Luyten et al., 2020). Also, consistent with the notion that attachment to parents/caregivers are an essential factor for time perspective (e.g., Akirmak, 2014), several studies show that stronger attachment to parents in adolescence is associated with higher scores on the positively valenced facets of ZTPI, and lower scores on Past Negative, Future Negative as well as Present Fatalistic (e.g., Blomgren et al., 2016; Laghi et al., 2016). By contrast, maladaptive attachment styles involving anxiety and avoidance in romantic relationships were associated with greater DBTP scores (Akirmak, 2014).

Mentalization capabilities were moreover shown to have a positive relation to the personality trait agreeableness (Allen et al., 2017), whereas higher DBTP was negatively associated with this trait (Bajec, 2018). Prosocial behaviors usually predominant in agreeableness personality trait is theorized to be promoted by a secure attachment, since secure relationships create an optimal environment for the development of emotional understanding (Costa et al., 2022). Thus, based on evidence of common links to attachment, and the idea that mentalization and time perspective could reinforce one another, one might expect that difficulties with mentalization are associated with greater deviations from the balanced time perspective.

So far, only one prior study that we know of (Wu et al., 2022), examined the association of measures mentalization and time perspective. The focus of the study was on evaluating the validity of the self-report questionnaire for mentalization they had developed rather than to examine these associations specifically, and the only hypothesis stated was that scores on Future might be expected to be associated with genuine mentalization. Their measure of mentalization (IMQ) contained two subscales related to mentalization. The first subscale (IMQ_SO) was intended to capture mentalization of others’ mental states from the perspective of the self, whereas the second subscale (IMQ_SS) aimed to capture the self-generated mental states from the perspective of the self. A third scale was developed to assess mentalization of own mental state from viewpoint of others, which they labeled as a “meta-metacognitive” aspect.

Focusing on the first two scales, tapping aspects usually regarded as part of the mentalization construct, the self-self (meta-cognition) scale showed results that were in line with the hypothesis outlined above, namely a significant positive correlation with Future in two separate samples (mean *r* = 0.33). Past Positive was in addition significantly associated with mentalization in both samples (mean *r* = 0.25). Finally, mentalization ability showed a significant negative association with Present Fatalistic, in one of the samples at least. The results for the second (self-to-others) scale showed a different pattern. In the latter case, greater scores on both ZTPI dimensions involving present time frame (Present Fatalistic, and also Present Hedonistic) were namely related to *better* perceived ability to judge other’s mental states, and also this scale was unrelated to scores on Future. Thus, part of the results by Wu et al. (2022) were consistent with the expectations that good mentalization ability should be paired with a more “BTP like” ZTPI score profile, for example lower score on Present Fatalistic and higher scores on Future, while other aspects of the results seem inconsistent with this prediction.

### The present study

Given the sparse evidence on the relation of time perspective and mentalization and the somewhat mixed results in Wu et al. (2022), we deemed a conceptual replication study motivated. We expanded on the prior study by adopting the six-factor version of the Zimbardo Time Perspective inventory (Carelli et al., 2011). Given that the new Future Negative scale was shown to be associated with anxiety, depression, and lower life satisfaction, we expected this scale to be related to lower levels of mentalization (hypomentalization). Finally, in addition to subscale analyses by Wu et al., we considered the DPBT measure described above. Under the assumption that this provides the best single measure of deviations from the healthy balanced profile we expected that this measure would show a particularly strong relationship with mentalization.

As a measure of mentalization we chose the Reflective Functioning Questionnaire (RFQ-8) by Fonagy et al. (2016). RFQ-8 is a widely used measure that showed good levels of internal consistency and was able to discriminate, for example, individuals with ADHD or borderline personality disorder from a healthy control group (Perroud et al., 2017). However, several recent studies (e.g., Müller et al., 2022; Woźniak-Prus et al., 2022; Horváth et al., 2023) questioned the validity of separate scales for certainty (hypermentalization; i.e. a tendency to make assumption that go beyond what is reasonable, i.e., feeling of knowing exactly what others think) vs. uncertainty (hypomentalization; i.e. an incapability, or reluctance, to think in nuanced perspectives about mental state of others, i.e., making interpretations in concrete and superficially observable ways) that were proposed by the developers. As noted by the authors of the latter articles, the fact that four out of the eight items are double coded and used as indicators both of hypo- and hypermentalization is problematic from viewpoint of the assumption of uncorrelated measurement errors. Also, scores on the hypermentalization scale, contrary to what would be expected from theory, were positively rather than negatively associated with indices of health in several of these studies. Finally, confirmatory factor analyses provided support of a unitary model. Given this matter, a first and necessary step in the current study was to identify a best fitting model for the version of RFQ-8 (in Swedish) that we used.

## Method

### Participants

A total of 258 individuals took part in the study (192 women, 62 men, 4 “other,” *M* = 30.4 years, *SD* = 11.4). The sample was a convenience sample recruited using flyers at Umeå University, links to the web survey in student groups and via Facebook pages. A majority (*n* = 163) were students, 71 were full- or part time employed, 4 were unemployed, 4 were on parental leave, 5 were on sick leave, and 11 marked “other” as occupation. As of health care status, 196 participants stated having no ongoing contact with health care system for some sort of psychological issue, 60 participants stated having an ongoing contact with health care system for some sort of psychological issue, and 2 participants did not want to specify health care status. All participants provided informed consent before filling out the form.

### Procedure

The measures of mentalization and time perspective were part of a web survey that was created using Microsoft Forms. The survey additionally contained questions regarding sex, age, occupation, and whether the participant had an ongoing contact with the health care system, for any type of psychological condition at present moment, or not.

### Measures

#### Swedish Zimbardo time perspective inventory

As the measure of time perspective, we used a short version of the Swedish Zimbardo Time Inventory (S-ZTPI, Carelli and Olsson, 2015; Molinari et al., 2016) that consists of 30 items, five each for the six dimensions; Past Positive, Past Negative, Present Hedonistic, Present Fatalistic; Future Positive and Future Negative. Each of the 30 statements are rated on a five-point scale: 1 = very untrue of me, 2 = somewhat untrue of me, 3 = neither true nor untrue of me, 4 = somewhat true of me and 5 = very true of me. Estimates of internal consistency in the present sample were as follows: Cronbach’s α PP (Past Positive) = 0.77, PF (Present Fatalistic) = 0.63, PP = 0.77, PN = 0.86, PH = 0.80, FP = 0.76, FN = 0.73.

DBTP was computed using following formula:


$$=\sqrt{\begin{array}{l} {\left(oPN-ePN\right)}^{2}+{\left(oPP-ePP\right)}^{2}+{\left(oPF-ePF\right)}^{2}+\\ {\left(oPH-ePH\right)}^{2}+{\left(oFP-eFP\right)}^{2}+{\left(oFN-eFN\right)}^{2} \end{array}}$$

Where PN = Past Negative, PP = Past Positive, PF = Present Fatalistic, PH = Present Hedonistic, FP = Future Positive, FN = Future Negative. Empirical, values (*e*) are the individuals’ mean score on each of the six dimensions. Optimal values (*o*) for ZTPI were adopted from Jankowski et al. (2020); PN = 1, PP = 5, PF = 1, PH = 3.4, FP (F) = 5, and in analogy with the reasoning in Jankowski et al. the optimal value for FN was set to 1.

#### Reflective functioning questionnaire

RFQ-8 (Fonagy et al., 2016; A Swedish version provided by the authors https://www.ucl.ac.uk/psychoanalysis/research/reflective-functioning-questionnaire-rfq was used) is a measure of mentalization that consists of eight statements that are rated on a Likert scale ranging from 1 = completely disagree to 7 = completely agree. The RFQ-8 is in the original study further divided into two subscales: Certainty about mental states (RFQc) and Uncertainty about mental states (RFQu). RFQc is intended to measure hypermentalizing and RFQu is intended to measure hypomentalizing. RFQc contains questions such as *“Peoples thoughts are a mystery to me,”* where low agreement yields a higher score on RFQc subscale and indicates larger degree of hypermentalizing. High agreement yields a lower score on RFQc subscale and indicates more genuine mentalizing. RFQu contains questions such as *“Strong feelings often cloud my thoughts.”* High agreement yields a higher score on the RFQu subscale and indicates larger degree of hypomentalizing. Low agreement yields a lower score on RFQu subscale and indicates more genuine mentalizing. Of the total 8 items of RFQ, 4 items are double scored and generate points on both subscales, whereas 2 items are specific for respective subscale. Scoring on either extreme on the Likert scale yield 1 to 3 points [3 2 1 0 0 0 0] or [0 0 0 0 1 2 3] for RFQc and RFQu, respectively (Fonagy et al., 2016). As an alternative, the RFQ items have been regarded to reflect a single dimension (ranging from genuine mentalization to hypomentalization), where all of the eight items (except item 7, “I always know what I feel” which needs to be reversed as a higher score indicates certainty of lower hypomentalization) are averaged to provide an overall score (e.g., Woźniak-Prus et al., 2022), whereas another study (Horváth et al., 2023) suggests that item 7 should be discarded before computing the composite score. Scoring could consider the entire range of responses to items (1–7 scoring) or, as suggested in the original study, recoding to 0-3 (0–3 scoring).

### Statistical methods

Statistical Analyses were conducted using IBM SPSS Statistics, SPSS AMOS version 29. For manifest variables Pearson’s correlations were computed. CFA/structural equation models were used to compare competing models of RFQ-8 and to examine correlations of latent (mentalization) and time perspective variables. At this point we used deviations calculated separately for the past, present, and future frames of the S-ZTPI as indicators of a common DBTP construct (Rönnlund and Carelli, 2018). For these analyses we considered three indices of model fit. The first was the chi-square denominator (x^2^/df) with a value below 2 being considered as good and a value below 3 acceptable. The second was Comparative Fit Index (CFI) for which a value of 0.95 or higher is desirable (Hu and Bentler, 1999), while 0.90 is often taken to indicate reasonable fit (e.g., Schumacker and Lomax, 2010). The third was the Root Mean Square Error of Approximation (RMSEA). Values for RMSEA at or below 0.06 indicate good model fit (Hu and Bentler, 1999), while values between 0.06 and 0.08 indicate reasonable fit (Browne and Cudeck, 1993).

## Results

**Figure 1:**
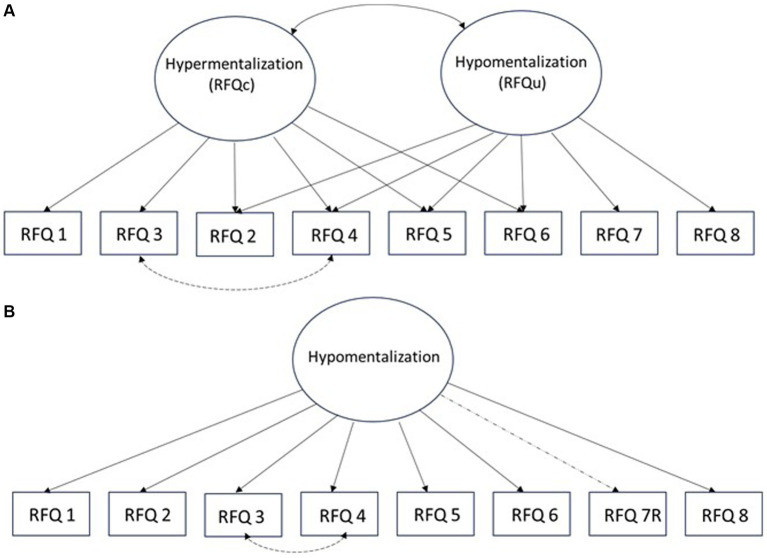
Outline of competing models of the latent structure of the RFQ-8 involving separate hyper- and hypomentalization factors **(A)** or **(B)** a single factor (hypomentalization). Correlated error terms for items 3 and 4 is based on prior studies (e.g., Ruiz-Parra et al., 2023).

We first set out to identify the best fitting model of the latent structure of RFQ-8. The competing models are depicted in simplified form in Figure 1. The first model is a two-factorial model concordant with the original model proposed by the developers. In this model, two items load exclusively on the Uncertainty (Hypomentalization) factor and two items exclusively on the Certainty (Hypermentalization) factor, whereas four of the items are assumed to load on both factors.

The two-factor model using 1–7 scoring of the items did not yield a permissible solution due to a case of negative error variance. Addition of correlated error term for items 3 and 4, done in prior studies, resulted in good values for the fit indices, but four item loadings were below 0.30 and non-significant (*p* > 0.05). A similar pattern of non-significant loadings was observed using 0-3 scoring. By contrast, the unitary model showed reasonable or good fit as deemed by the indices; chi/2 = 2.29, CFI = 0.96, RMSEA = 0.071, and even better fit based on 0-3 scoring, x^2^/df = 1.97, CFI = 0.96, RMSEA = 0.061. Removing item 7, as suggested by one study, did not improve, but rather decrease, the model fit. Thus, we retained the unitary model involving the eight RFQ items.

Given the adequate fit of the unitary model and evidence that the measure mainly reflects hypomentalization (uncertainty), we computed a mean RFQ-8 score based on the 0-3 scoring method of the items. The internal consistency of the scale was acceptable (α = 0.76). Next, we computed Pearson’s correlations of the main study variables. The results together with descriptive statistics for the measures are presented in Table 1. The results of primary interest, namely correlations of RFQ-8 total score and each of the time perspective measures are provided in the first column. As we can see, the measure of (hypo)mentalization was positively associated with each of the negative time perspective dimensions, including Past Negative, Present Fatalistic, and Future Negative (*r*s = 0.41–0.47). Additionally, a statistically significant positive association with Present Hedonistic (*r* = 0.31) was observed. Moreover, as expected, RFQ was inversely associated with Future Positive.

**Table 1:** Zero-order correlations and descriptives of the variables in the study.

Measure	1	2	3	4	5	6	7	8
RFQ-8	1	0.06						
Past positive	0.00	1						
Past negative	0.47***	−0.26***	1					
Present hedonistic	0.31***	0.14*	0.17**	1				
Present fatalistic	0.41***	0.04	0.26***	0.29***	1			
Future positive	−0.29***	0.11	−0.16*	−0.14*	−0.19**	1		
Future negative	0.43***	−0.15*	0.59***	0.16*	0.36***	−0.32***	1	
DBTP	0.49***	−0.49***	0.78***	0.08	0.45***	−0.51***	0.76***	1
*M*	0.89	3.51	3.01	2.73	2.43	3.35	2.65	4.11
*SD*	0.56	0.77	0.99	0.82	0.65	0.74	0.80	1.09
Skewness	0.72	−0.60	0.07	0.29	0.28	−0.34	0.28	0.23
Kurtosis	−0.03	0.33	−0.80	−0.40	−0.29	−0.21	−0.60	0.15

*p <.05, **p < .01, ***p < .001.

Of major interest, RFQ-8 showed a positive association with DBTP (*r* = 0.49). Neither age nor gender or occupational status were associated with any of the two variables (*p*s > 0.10). However, contact with health care for some psychological issue was associated with higher RFQ-8 (*r* = 0.21, *p* < 0.001) as well as DBTP (*r* = 0.37, *p* < 0.001). The magnitude of the association of RFQ and DBTP was only marginally different from the zero-order correlation when this variable was adjusted for (partial correlation) though (*r* = 0.45, *p* < 0.001).

Even though the correlation of RFQ and DBTP was in the high moderate range it is likely an underestimate of the true association of the construct due to measurement error. To test for this, we set up a model including both constructs as latent variables [i.e., the unitary model of RFQ-8 and with deviation scores for each of the time frames (i.e., past, present, future) as the indicators of the DBTP construct].

**Figure 2:**
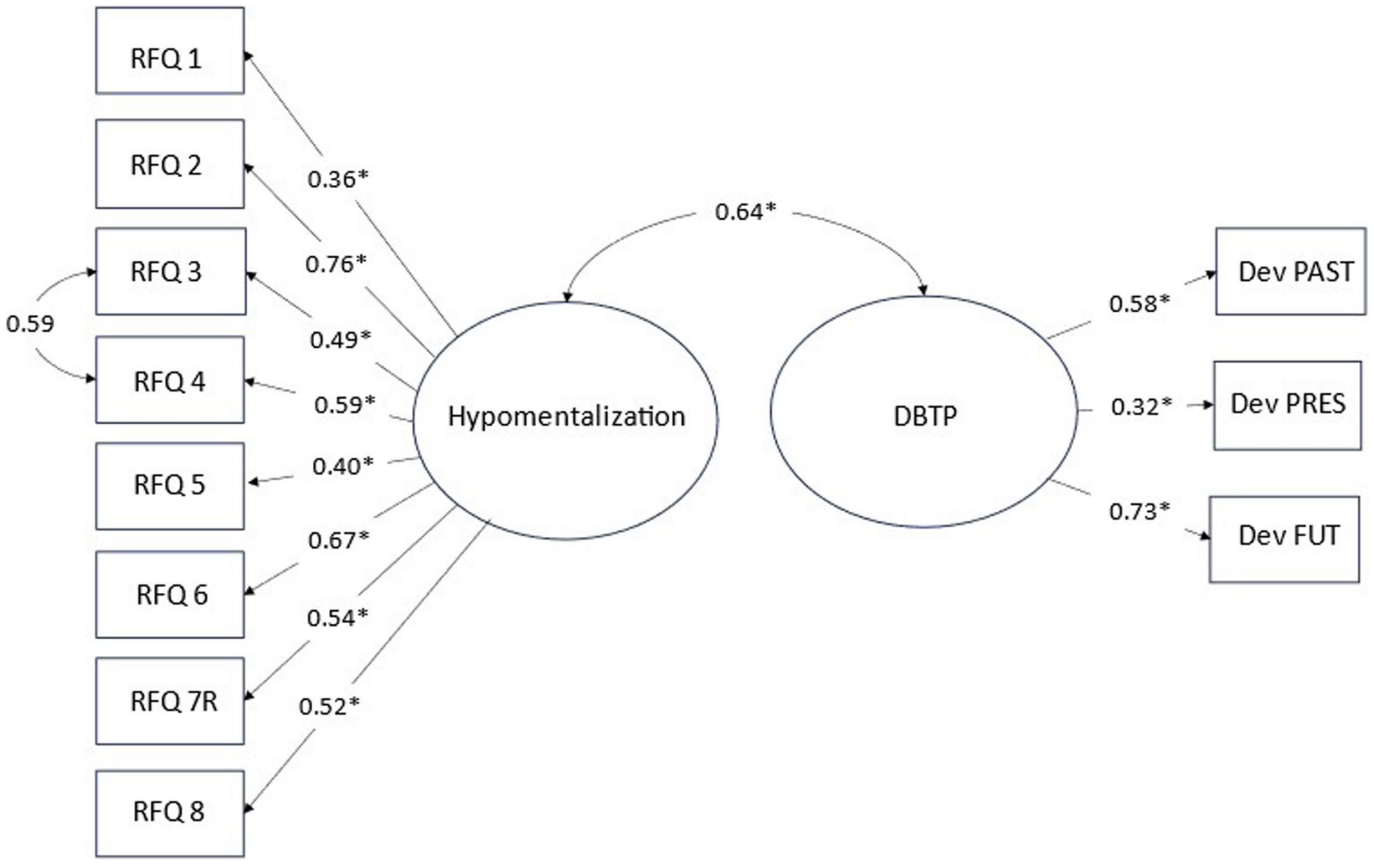
Structural model of hypomentalizing and DPT, including correlation of construct and item loadings. *p < 0.01.

The model including item loadings based on data for the entire sample is summarized in Figure 2. The model fit was reasonable as judged by the three indices, x^2^/df = 2.38, CFI = 0.901, and RMSEA = 0.073. Of primary interest, the result indicated a strong positive association (*r* = 0.64) of the two latent constructs, such that greater deviations from the balanced time perspective are associated with a higher degree of hypomentalization. A model where contact with health care for a psychological issue (binary coded; *n* = 256) was as a predictor of both latent variables to adjust for this potential confound yielded a very similar estimate (*r* = 0.62).

## Discussion

The aim of the current study was to examine the relationship between mentalization measured by a version of the Reflective Functioning Questionnaire (Fonagy et al., 2016), and time perspective as operationalized by Zimbardo and Boyd (1999) and Carelli et al. (2011). Initial analyses favored a unitary model of RFQ-8, which adds to the evidence that a single factor reflecting hypomentalization is sufficient to account for covariation of the items in this questionnaire (e.g., Horváth et al., 2023; Müller et al., 2022; Woźniak-Prus et al., 2022; Ruiz-Parra et al., 2023).

In line with our hypothesis and consistent with aspects of the results in the prior study by Wu et al. (2022), ZTPI dimensions generally associated with less adaptive behaviors, anxiety, and depression—i.e., Past Negative and Present Fatalistic—were associated with higher hypomentalization. By contrast, the Future (Positive) dimension showed a statistically significant inverse relationship to hypomentalization, while Past Positive, contrary to what we expected, and contrary to Wu et al., showed no such relationship. Expanding on the study by Wu et al., that involved a single Future dimension, we furthermore found that Future Negative showed a rather substantial positive association with hypomentalization. Most importantly, the current study demonstrated a strong association of DBTP, summarizing biases across the entire ZTPI score profile, and hypomentalization, suggesting that poor mentalization skills (hypomentalization) and the degree of overall time perspective biases are substantially related.

While our results showed several points of convergence with results for the self-to-self scale in Wu et al. (2022), their results for the self-to-other scale described earlier differed substantially from the present findings and their own results on the self-to-self scale. RFQ-8 is a global measure of mentalization where, as noted by others, most items concern reflection on one’s own thoughts and feelings, rendering it difficult to speculate on the divergent finding in the prior study. Future studies could, for example, use an expanded version of RFQ that allows for separating mentalizing self- vs. others (Derks et al., 2023; for an alternative self-report measure, see Müller et al., 2023) to determine whether associations with aspects of time perspective differ across these two polarities.

Besides the need to examine the generalizability of results to other-focused mentalization, further research is required to identify the factors that account for the co-variation of mentalization and DBTP. We mentioned attachment style as one potential factor and the viability of this account needs to be tested. Another factor to consider is mindfulness, defined as “the awareness that arises through paying attention, on purpose, in the present moment, non-judgmentally” (Kabat-Zinn, 1990). More specifically, the ability to focus on the here-and-now with an accepting mind has been considered a gateway to greater understanding of self and others, and a significant positive association of mentalization and mindfulness was observed in prior studies involving non-clinical samples (Török and Kéri, 2022; Ruiz-Parra et al., 2023). Similarly, higher trait mindfulness was associated with smaller DBTP values (Stolarski et al., 2016; Rönnlund et al., 2019), and mindfulness-based interventions significantly decreased DBTP (Rönnlund et al., 2019), presumably because of greater acceptance of the present may generalize to the past and reduces tendencies to ruminate on past experiences and worry about the future.

A related construct that might be explored in this context is psychological flexibility. Psychological flexibility is defined as the ability to stay in contact with the present moment, regardless of unpleasant thoughts, feelings, and sensations, while choosing and developing one’s behavior repertoire based on personal values and situational contexts (Hayes et al., 1999). By contrast, inflexibility is manifested for example as experiential avoidance of certain thoughts, feelings, and sensations—a tendency that may provide short-term gains (and thus be reinforced) but have negative long-term consequences. Psychological inflexibility showed a strong positive association with DBTP (Pyszkowska and Rönnlund, 2021). Presumably, a high degree of experiential avoidance, lacking in openness and distance to one’s own thoughts, could narrow one’s repertoire of experiences rendering understanding one’s thoughts, feelings, and behaviors more difficult to understand, something which could be examined in a future study.

### Limitations

Despite strengths in form of use of well researched measures of the main constructs and attention to latent factors, the present study has limitations. Apart from the limitation of components of mentalization measured (e.g., little focus on mentalizing others), the sole use of questionnaires to assess the constructs may induce share method variance. At least for mentalization, behavioral approaches are available that might be considered in future studies.

## Conclusion

In conclusion, the present research enforces and extends the finding of significant relationships between facets of time perspective and mentalization in a prior study, and demonstrated a strong association of deviations from the optimal time perspective profile and hypomentalization. Future research is required to examine the generalizability to measures capturing mentalization of others and to consider variables that might account for the shared variance.

## Data availability statement

The raw data supporting the conclusions of this article will be made available by the authors, without undue reservation.

## Ethics statement

The studies involving humans were approved by Etikprövningsmyndigheten, Sverige. The studies were conducted in accordance with the local legislation and institutional requirements. The participants provided their written informed consent to participate in this study.

## Author contributions

AW: Conceptualization, Formal analysis, Investigation, Methodology, Writing – original draft. MR: Conceptualization, Data curation, Formal analysis, Methodology, Writing – review & editing.

## References

[ref1] Akirmak U. (2014). How is time perspective related to perceptions of self and interpersonal relationships? Span. J. Psychol. 17, 1–11. doi: 10.1017/sjp.2014.92 26055809

[ref2] Allen J. Fonagy P. (2007). Handbook of mentalization-based treatment. Chichester: Wiley and Sons Ltd.

[ref3] Allen T. A. Rueter A. R. Abram S. V. Brown J. Deyoung C. G. (2017). Personality and neural correlates of Mentalizing ability. Eur. J. Personal. 31, 599–613. doi: 10.1002/per.2133, PMID: 29610548 PMC5877481

[ref4] Åström E. Seif A. Wiberg B. Carelli M.-G. (2019). Getting “stuck” in the future or the past: relationships between dimensions of time perspective, executive functions, and repetitive negative thinking in anxiety. Psychopathology 51, 362–370. doi: 10.1159/000494882, PMID: 30522113

[ref5] Bajec B. (2018). Relationship between time perspective and job satisfaction. Int. J. Hum. Resourc. Develop. Manag. 8, 145–163. doi: 10.1504/IJHRDM.2018.092294

[ref6] Bateman A. Fonagy P. (2016). What is mentalizing? Mentalization-based treatment for personality disorders: A practical guide. Oxford: Oxford Academic.

[ref7] Blomgren A.-S. Svahn K. Åström E. Rönnlund M. (2016). Coping strategies in late adolescence: relationships to parental attachment and time perspective. J. Genet. Psychol. 177, 85–96. doi: 10.1080/00221325.2016.1178101, PMID: 27177122

[ref8] Browne M. W. Cudeck R. (1993). “Alternative ways of assessing model fit” in Testing structural equation models. eds. Bollen K. A. Long J. S. (Beverly Hills, CA: Sage), 136–162.

[ref9] Carelli M. G. Olsson C. J. (2015) Neural correlates of time perspective. In: Stolarski M Fieulain N Beek W van (Eds), Time perspective theory: Review, research and application. Essays in honor of Philip Zimbardo, Cham, Switzerland: Springer International Publishing, 231–242

[ref10] Carelli M. G. Wiberg B. (2012). Time out of mind: temporal perspective in adults with ADHD. J. Atten. Disord. 16, 460–466. doi: 10.1177/1087054711398861 21490173

[ref11] Carelli M. G. Wiberg B. Wiberg M. (2011). Development and construct validation of the Swedish Zimbardo time perspective inventory (S-ZTPI). Eur. J. Psychol. Assess. 27, 220–227. doi: 10.1027/1015-5759/a000076

[ref12] Chung Y. S. Barch D. Strube M. (2014). A meta-analysis of mentalizing impairments in adults with schizophrenia and autism spectrum disorder. Schizophr. Bull. 40, 602–616. doi: 10.1093/schbul/sbt048, PMID: 23686020 PMC3984506

[ref13] Costa M. M. Santos C. Fernandes M. Veríssimo M. (2022). Attachment and the development of prosocial behavior in children and adolescents: a systematic review. Children 9:874. doi: 10.3390/children9060874, PMID: 35740811 PMC9222107

[ref14] Derks S. D. M. Willemen A. M. Vrijmoeth C. Sterkenburg P. S. (2023). Lessons learned from the adaptation of the reflective functioning questionnaire (RFQ) for Dutch people with mild to borderline intellectual disabilities. PLoS One 18:e0287751. doi: 10.1371/journal.pone.0287751, PMID: 37368894 PMC10298756

[ref15] Fischer-Kern M. Tmej A. (2019). Mentalization and depression: theoretical concepts, treatment approaches and empirical studies - an overview. Z. Psychosom. Med. Psychother. 65, 162–177. doi: 10.13109/zptm.2019.65.2.162, PMID: 31154932

[ref16] Fonagy P. Luyten P. Moulton-Perkins A. Lee Y. W. Warren F. Howard S.. (2016). Development and validation of a self-report measure of mentalizing: the reflective functioning questionnaire. PLoS One 11:e0158678. doi: 10.1371/journal.pone.0158678, PMID: 27392018 PMC4938585

[ref17] Hayes S. Strosahl K. D. Wilson K. G. (1999). Acceptance and commitment therapy: Understanding and treating human suffering. New York, NY: Guilford Press.

[ref18] Horváth Z. Demetrovics O. Paksi B. Unoka Z. Demetrovics Z. (2023). The reflective functioning questionnaire-revised- 7 (RFQ-R-7): a new measurement model assessing hypomentalization. PLoS One 18:e0282000. doi: 10.1371/journal.pone.0282000, PMID: 36827243 PMC9956064

[ref19] Hu L. Bentler P. M. (1999). Cutoff criteria for fit indexes in covariance structure analysis: conventional criteria versus new alternatives. Struct. Equ. Model. 6, 1–55. doi: 10.1080/10705519909540118

[ref21] Jankowski K. S. Zajenkowski M. Stolarski M. (2020). What are the optimal levels of time perspectives? Deviation from the balanced time perspective-revisited (DBTP-r). Psychol. Belgica 60, 164–183. doi: 10.5334/pb.487, PMID: 32607249 PMC7319069

[ref22] Kabat-Zinn J. (1990). Full catastrophe living: How to cope with stress, pain and illness using mindfulness meditation. New York, NY: Bantam Dell.

[ref23] Laghi F. Pallini S. Baumgartner E. Guarino A. Baiocco R. (2016). Parent and peer attachment relationships and time perspective in adolescence: are they related to satisfaction with life? Time Soc. 25, 24–39. doi: 10.1177/0961463X15577282

[ref24] Lewin K. (1951). Field theory in the social sciences: selected theoretical papers. New York: Harper.

[ref25] Luyten P. Campbell C. Allison E. Fonagy P. (2020). The mentalizing approach to psychopathology: state of the art and future directions. Annu. Rev. Clin. Psychol. 16, 297–325. doi: 10.1146/annurev-clinpsy-071919-015355, PMID: 32023093

[ref26] Molinari L. Speltini G. Passini S. Carelli M. G. (2016). Time perspective in adolescents and young adults: enjoying the present and trusting in a better future. Time Soc. 25, 594–612. doi: 10.1177/0961463X15587833

[ref27] Müller S. Wendt L. P. Spitzer C. Masuhr O. Back S. N. Zimmermann J. (2022). A crucial evaluation of the reflective functioning questionnaire (RFQ). J. Pers. Assess. 104, 613–627. doi: 10.1080/00223891.2021.1981346 34597256

[ref28] Müller S. Wendt L. P. Zimmermann J. (2023). Development and validation of the certainty about mental states questionnaire (CAMSQ): a self-report measure of mentalizing oneself and others. Assessment 30, 651–674. doi: 10.1177/10731911211061280, PMID: 34905983 PMC9999289

[ref29] Papastamatelou J. Unger A. Giotakos O. Athanasiadou F. (2015). (2015). Is time perspective a predictor of anxiety and perceived stress? Some preliminary results from Greece. Psychol. Stud. 60, 468–477. doi: 10.1007/s12646-015-0342-6

[ref30] Perroud N. Badoud D. Weibel S. Nicastro R. Hasler R. Küng A.. (2017). Mentalization in adults with attention deficit hyperactivity disorder: comparison with controls and patients with borderline personality disorder. Psychiatry Res. 256, 334–341. doi: 10.1016/j.psychres.2017.06.087, PMID: 28675858

[ref31] Pyszkowska A. Åström E. Rönnlund M. (2024). Deviations from the balanced time perspective, cognitive fusion, and self-compassion in individuals with or without a depression diagnosis: different mean profiles but common links to depressive symptoms. Front. Psychol. 14:1290676. doi: 10.3389/fpsyg.2023.1290676, PMID: 38250112 PMC10796795

[ref32] Pyszkowska A. Rönnlund M. (2021). Psychological flexibility and self-compassion as predictors of well-being: mediating role of a balanced time perspective. Front. Psychol. 12:671746. doi: 10.3389/fpsyg.2021.671746, PMID: 34177730 PMC8222535

[ref33] Rönnlund M. Åstrom E. Adolfsson R. Carelli M. G. (2018). Perceived stress in adults aged 65 to 90: relations to facets of time perspective and COMT Val158Met polymorphism. Front. Psychol. 9:378. doi: 10.3389/fpsyg.2018.00378, PMID: 29623060 PMC5874313

[ref34] Rönnlund M. Åström E. Carelli M. G. (2017). Time perspective in late adulthood: aging patterns in past, present and future dimensions, deviations from balance, and associations with subjective well-being. Timing Time Percept. 5, 77–98. doi: 10.1163/22134468-00002081

[ref35] Rönnlund M. Carelli M. G. (2018). Deviations from a balanced time perspective in late adulthood: associations with current g and g in youth. Intelligence 71, 8–16. doi: 10.1016/j.intell.2018.09.002

[ref36] Rönnlund M. Koudriavtseva A. Germundsjö L. Eriksson T. Åström E. Carelli M. G. (2019). Mindfulness promotes a more balanced time perspective: correlational and intervention-based evidence. Mindfulness 10, 1579–1591. doi: 10.1007/s12671-019-01113-x

[ref37] Ruiz-Parra E. Manzano-García G. Mediavilla R. Rodríguez-Vega B. Lahera G. Moreno-Pérez A. I.. (2023). The Spanish version of the reflective functioning questionnaire: validity data in the general population and individuals with personality disorders. PLoS One 18:e0274378. doi: 10.1371/journal.pone.0274378, PMID: 37023214 PMC10079014

[ref38] Schumacker R. E. Lomax R. G. (2010). A beginner’s guide to structural equation modeling. 3rd Edn. New York, NY: Routledge Academic.

[ref39] Sloover M. van Est L. A. C. Janssen P. G. J. Hilbink M. van Ee E. (2022). A meta-analysis of mentalizing in anxiety disorders, obsessive-compulsive disorder and related disorders, and trauma and stressor related disorders. J. Anxiety Disord. 92:102641. doi: 10.1016/j.janxdis.2022.102641, PMID: 36257080

[ref40] Stolarski M. Bitner J. Zimbardo P. G. (2011). Time perspective, emotional intelligence, and discounting of delayed awards. Time Soc. 20, 346–363. doi: 10.1177/0961463X11414296

[ref41] Stolarski M. Vowinckel J. Jankowski K. S. Zajenkowski M. (2016). Mind the balance, be contented: balanced time perspective mediates the relationship between mindfulness and life satisfaction. Personal. Individ. Differ. 93, 27–31. doi: 10.1016/j.paid.2015.09.039

[ref42] Stolarski M. Zajenkowski M. Jankowski K. S. Szymaniak K. (2020). Deviation from the balanced time perspective: a systematic review of empirical relationships with psychological variables. Personal. Individ. Differ. 156, 109772–109719. doi: 10.1016/j.paid.2019.109772

[ref43] Styła R. Stolarski M. Szymanowska A. (2019). Linking childhood adversities with schizophrenia: a mediating role of the balanced time perspective. Schizophr. Res. 209, 281–283. doi: 10.1016/j.schres.2019.05.021, PMID: 31126804

[ref44] Török E. Kéri S. (2022). The relationships among mentalization, mindfulness, working memory, and schizotypical personality traits in the general population. Front. Psychol. 13:682889. doi: 10.3389/fpsyg.2022.682889, PMID: 35586232 PMC9108540

[ref45] Woźniak-Prus M. Gambin M. Cudo A. Sharp C. (2022). Investigation of the factor structure of the reflective functioning questionnaire (RFQ-8): one or two dimensions? J. Pers. Assess. 104, 736–746. doi: 10.1080/00223891.2021.2014505 35015610

[ref46] Wu H. Fung B. J. Mobbs D. (2022). Mentalizing during social interaction: the development and validation of the interactive mentalizing questionnaire. Front. Psychol. 12:791835. doi: 10.3389/fpsyg.2021.791835, PMID: 35250692 PMC8891136

[ref47] Zimbardo P. G. Boyd J. N. (1999). Putting time in perspective: a valid, reliable individual-differences metric. J. Pers. Soc. Psychol. 77, 1271–1288. doi: 10.1037/0022-3514.77.6.1271

[ref48] Zimbardo P. Boyd J. (2008). The time paradox: The new psychology of time that will change your life. New York: Simon and Schuster.

